# Supplementary material for: The effect of dietary diversity on anemia levels among children 6–23 months in sub-Saharan Africa: A multilevel ordinal logistic regression model
Source: PLoS One. 2024 May 21;19(5):e0298647. doi: 10.1371/journal.pone.0298647 (PMC11108208; doi:10.1371/journal.pone.0298647)
Supplement: Attachment — Submitted filename: Reviewer Comments_PONE-D-23-21819.docx [file pone.0298647.s002.docx]

**Reviewer Comments**

This is an interesting study on the association between dietary diversity and anaemia among children. Proper dietary balance is crucial for anaemia particularly for children as this may interfere with their growth and development.

I propose to address the following issues.

Abstract:

Line 61: Though child undernutrition like stunting, wasting, and underweight is crucial to address early, these are not the focal point of this research. Therefore, in my opinion it is not necessary to mention this here.

Introduction:

Line 88: ID need to be abbreviated at first.

Methodology:

Line 209: It was also better to perform binary logistic regression analysis converting anaemia as a binary variable (anaemic vs non-anaemic)

Results:

Line 277: Reference need to be corrected.

Line 282: Reference need to be corrected.

Discussion:

The articles focal point is the relation between MDD and anaemia among children. However, in the discussion part little has been discussed about this. Other secondary factors have been discussed in detail. In my opinion, the author should need to focus on MDD and anaemia, rather than on other factors.

General Comments:

The research was done to understand the relationship between MDD and anaemia among children in SSA. This is a nice study with large sample size covering multiple countries in Africa. The author needs to be very specific in focusing the relationship between MDD and anaemia.
