# Supplementary material for: The effect of dietary diversity on anemia levels among children 6–23 months in sub-Saharan Africa: A multilevel ordinal logistic regression model
Source: PLoS One. 2024 May 21;19(5):e0298647. doi: 10.1371/journal.pone.0298647 (PMC11108208; doi:10.1371/journal.pone.0298647)
Supplement: Attachment — Submitted filename: Reviewer report_plosOne.docx [file pone.0298647.s003.docx]

As the reviewer, I have conducted a detailed assessment of the manuscript titled "The effect of dietary diversity on anemia levels among children 6-23 months in Sub-Saharan Africa: A multilevel ordinal logistic regression model" using the STROBE Statement checklist for observational studies. Here is an in-depth report addressing each of the checklist items and providing specific line references where issues have been identified:

**Title and Abstract:**

1. **(a)** The title accurately indicates the study's design as a multilevel ordinal logistic regression model.
   - No issues identified.
2. **(b)** The abstract effectively describes what was done but falls short in presenting the study's findings in relation to the research questions. The study aimed to determine the association between dietary diversity and anemia prevalence, but the abstract separates these variables. Consequently, the results fail to demonstrate an association between MDD and reduced anemia prevalence, leading to a potentially misleading conclusion (Lines 27-32).

**Introduction:**

2. The introduction section presents several issues:
   - Line 75 should refer to "g/dL" instead of "g/d."
   - Lines 79-82 contain repetitive information regarding the effects of anemia on growth and development, which should be restructured.
   - There is a repetitive discussion of the causes of anemia in lines 86-91 and 92-95, making it monotonous to read.
   - The introduction inadequately addresses the role of dietary diversity in anemia prevalence and lacks relevant literature to establish the rationale.
   - Line 111-122 is better suited for the discussion section, as its placement in the introduction is inappropriate. Overall, the introduction is poorly written.

**Objectives:**

3. The manuscript does not clearly state specific study objectives, which is a critical omission. Specific objectives should be explicitly defined, including any prespecified hypotheses.

**Methods:**

4. The manuscript accurately presents the key elements of the study design, appropriately considering it as a secondary data analysis.
5. The description of the setting, locations, and data collection dates is correctly provided.
6. Since this is a secondary data analysis, the absence of eligibility criteria and participant selection details is acceptable.
7. All dependent and predictor variables are well-defined.
8. While the sources of data for each variable are described, the method of data collection for minimum dietary diversity is not mentioned.
9. The manuscript describes efforts to address potential sources of bias, considering various variables that may influence anemia levels in children.
10. No sample size calculation is provided, but the distribution of sample size by survey is tabulated. It remains unclear if the reported 52,180 was the total sample for the 32 countries. This issue should be clarified to ensure the accuracy of sample size representation.
11. The manuscript explains how quantitative variables were handled in the analyses, including the use of different analytical techniques. However, irrelevant model types are described (Lines 209-215), which should be removed. Ethical considerations should be moved before the method section, following the study setting for better organization.
12. The statistical methods, including multilevel logistic regression to control for potential confounding variables, are well-described. However, it is not indicated how missing data were analyzed.

**Results:**

13. There are several issues in the results section:

- Discrepancies in participant numbers for different countries and missing data (Line 278, Table 2).
- There is discrepancies such as dietary diversity data being available for 746 individuals in Swaziland instead of 763, in Gambia 3527 instead of 1172, and in South Africa 867 in instead of 293. This is just few discrepancies as an example. Thus, the manuscript should clarify how the sample size for each country was determined and address the potential bias introduced by these variations.
- The reference error "Error ! Reference source not found" needs clarification.
- Missing data in baseline variables should be explained, along with the methods used to address the missing data.
- Lines 304 to 308 are better placed in the discussion section.
- Table 4 should include anemia vs. dietary diversity, and a subgroup analysis for different categories of anemia. It is recommended to perform a logistic regression model for anemia vs. MDD, adjusting for various socioeconomic confounders instead of relying on the chi-square results (Table 4).
- The manuscript should clarify the model fit statistics referenced in the text (Lines 313-323). It is not clear why different anemia levels are being compared.

**Discussion:**

18. The discussion should be refocused on the primary study objective, which is the association between anemia levels and MDD. The manuscript appears to emphasize individual significant results of secondary predictors instead.

- While the manuscript mentions the large sample size as a strength indicating there is adequate power to detect the true effect of the independent variable. However, it does not provide a power calculation to support this claim.
- Some limitations are discussed, but the limitation related to the 24-hour recall for minimum dietary diversity is not mentioned and there is potential social desirability bias that could impact on the results. Discuss finding should be interpreted with caution.

**Recommendation**

This comprehensive report outlines the issues identified throughout the manuscript, with specific line references and requires substantial revisions to address these concerns and enhance the clarity, relevance, and presentation of results. After making the necessary revisions, a reevaluation is recommended for potential publication.
