# Supplementary material for: The effect of dietary diversity on anemia levels among children 6–23 months in sub-Saharan Africa: A multilevel ordinal logistic regression model
Source: PLoS One. 2024 May 21;19(5):e0298647. doi: 10.1371/journal.pone.0298647 (PMC11108208; doi:10.1371/journal.pone.0298647)
Supplement: Attachment — Submitted filename: Response to Reviewers.docx [file pone.0298647.s004.docx]

**Reviewer 1: Comments with response**

Thank you for spending your golden time and providing constructive comments on this manuscript

**Abstract:**

Line 61: Though child under-nutrition like stunting, wasting, and underweight is crucial to address early, these are not the focal point of this research. Therefore, in my opinion it is not necessary to mention this here.

- Thank you for this important comment related to the reality, then by considering this comment we have adjust while preparing revised manuscript

**Introduction:**

Line 88: ID need to be abbreviated at first.

- Thank you for this important comment, then by considering this comment we have incorporate it while preparing revised manuscript

**Methodology:**

Line 209: It was also better to perform binary logistic regression analysis converting anaemia as a binary variable (anaemic vs non-anaemic)

- Thanks for this comment related to the nature of variable. The outcome variable (anaemia) can be categorized as anaemia (anaemic vs non-anaemic) or anaemia level (non-anaemic, mild, moderate, and severe anaemic). Even though one can perform binary logistic regression analysis converting anaemia as binary variable (anaemic vs non-anaemic), most of scholars or evidence suggest the nature of outcome variable (anaemic level) is ordinal. So in line with ranked nature of the outcome variable, we have perform ordinal logistic regression analysis converting anaemia level as ordinal (non-anaemic, mild, moderate, and severe anaemic).

**Results:**

Line 277: Reference need to be corrected.

- Thank you for your comment. We have incorporated this comment while preparing revised manuscript.

Line 282: Reference need to be corrected.

- Thank you for your comment. We have incorporated this comment while preparing revised manuscript.

**Discussion:**

The articles focal point is the relation between MDD and anaemia among children. However, in the discussion part little has been discussed about this. Other secondary factors have been discussed in detail. In my opinion, the author should need to focus on MDD and anaemia, rather than on other factors.

- Thank you for this significant comment related to reality, by taking this as a comment we have included this assessment on the revised manuscript after carefully referring the result.

**General Comments:**

The research was done to understand the relationship between MDD and anaemia among children in SSA. This is a nice study with large sample size covering multiple countries in Africa. The author needs to be very specific in focusing the relationship between MDD and anaemia.

**Reviewer 2: Comments and response**

Reviewer #2: The authors have conducted an analysis of predictors of anemia severity in diverse sub-Saharan African populations using the Demographic and Health Surveys. Overall the manuscript is quite well written. The methods, results and conclusions are described with clarity. The findings are novel. I just have some minor comments.

1. Table 5. I assume the AOR is the proportionate odds ratio. This should be clarified in the title or footnotes.
2. Please provide a description of "cut-points." It is not clear what this is referring to.
3. Page 23. There seem to be a problem with error associated with references. That should be fixable.
4. With regard to "cluster" (page 17), I assume the cluster is based on the Enumeration Area. Please clarify.

Otherwise, the paper adds significant knowledge to this issue.

**Response: #1,** Thank you for these comments. By considering this comment we have incorporates while preparing the revised manuscript.

**Response: #2, Cut-points** used to obtained the odd ratio results**.** The Brant test of parallel odds assumption showed that odds ratios appeared to have held constant across all cut-off points of childhood anemia status for the final model at a 5% significance level (p-value = 0.08). The interpretations of odds ratio results obtained by modeling severely anemic vs moderately/mild/non-anemic; and anemic vs non-anemic were the same.

**Response: #3,** Thanks for this comment and by considering this we have fixed while preparing revised manuscript.

**Response: #4,** Yes you are right and we have mentioned under data source and study setting as well.

**Reviewer 3: Comments and response**

Thanks for spending your golden time and providing constructive comments on this manuscript.

**Title and Abstract:**

1. **(a)** The title accurately indicates the study's design as a multilevel ordinal logistic regression model.
   - No issues identified.
2. **(b)** The abstract effectively describes what was done but falls short in presenting the study's findings in relation to the research questions. The study aimed to determine the association between dietary diversity and anemia prevalence, but the abstract separates these variables. Consequently, the results fail to demonstrate an association between MDD and reduced anemia prevalence, leading to a potentially misleading conclusion (Lines 27-32).

**Response:** Thanks for this helpful comment related to the reality, and then by considering this comment we have incorporated while preparing revised manuscript.

**Introduction:**

3. The introduction section presents several issues:
   - Line 75 should refer to "g/dL" instead of "g/d."
   - Lines 79-82 contain repetitive information regarding the effects of anemia on growth and development, which should be restructured.
   - There is a repetitive discussion of the causes of anemia in lines 86-91 and 92-95, making it monotonous to read.
   - The introduction inadequately addresses the role of dietary diversity in anemia prevalence and lacks relevant literature to establish the rationale.
   - Line 111-122 is better suited for the discussion section, as its placement in the introduction is inappropriate. Overall, the introduction is poorly written.

**Response:** Thank you very much for this constructive comments related to the issues under introduction**.** As you know under introduction, it is better to show the efforts made by the concerned body to address the issue and the gap we have tried to fill by conducting this study. So by considering this fact, we have incorporated all comments while preparing the revised manuscript.

**Objectives:**

4. The manuscript does not clearly state specific study objectives, which is a critical omission. Specific objectives should be explicitly defined, including any prespecified hypotheses.

**Response:** Thanks for this comment. But every journals have own **“*manuscript body formatting guidelines”***. So by considering the ***“manuscript body formatting guidelines”*** of “**PLOS ONE”** we have stated the objective under background.

**Methods:**

5. The manuscript accurately presents the key elements of the study design, appropriately considering it as a secondary data analysis.
6. The description of the setting, locations, and data collection dates is correctly provided.
7. Since this is a secondary data analysis, the absence of eligibility criteria and participant selection details is acceptable.
8. All dependent and predictor variables are well-defined.
9. While the sources of data for each variable are described, the method of data collection for minimum dietary diversity is not mentioned.
10. The manuscript describes efforts to address potential sources of bias, considering various variables that may influence anemia levels in children.
11. No sample size calculation is provided, but the distribution of sample size by survey is tabulated. It remains unclear if the reported 52,180 was the total sample for the 32 countries. This issue should be clarified to ensure the accuracy of sample size representation.
12. The manuscript explains how quantitative variables were handled in the analyses, including the use of different analytical techniques. However, irrelevant model types are described (Lines 209-215), which should be removed. Ethical considerations should be moved before the method section, following the study setting for better organization.
13. The statistical methods, including multilevel logistic regression to control for potential confounding variables, are well-described. However, it is not indicated how missing data were analyzed.

**Response:** Since the study was based on the most recent Demographic and Health Survey (DHS) data of 32 SSA countries**.** By considering this fact**,** we have incorporated while preparing the revised manuscript as follows:

**No (8):** Regarding minimum dietary diversity: When children aged 6–23 months receive four or more of the seven dietary groups, it is said to have MDD [48-50]. Along with breast milk, the children were required to eat from at least four of the seven food categories. The seven food classes were: cereals, roots, and tubers; legumes and nuts; dairy products (milk, yogurt, cheese); flesh foods (meat, fish, fowl, liver, or other organs); eggs; fruits and vegetables high in vitamin A; and other fruits and vegetables. The scores for each food group, which ranged from 0 to 7, were added up to estimate MDD. A score of one (1) was given to any child who consumed any of the food groups, and a score of zero (0) was given to any child who did not. Children who consumed at least four ($\geq4$) of the food groups were considered to have an adequate MDD, and this was coded as "1", whereas the remaining children who consumed fewer than four food groups were coded as "0 = inadequate". This classification and categorization was inspired by studies on either dietary diversity by itself or its association to under-nutrition [48-50]

**No (10):** Since the source of data is secondary data and then we extracted the data from the KR dataset based on literature and then appended using the STATA command “append using”. A sample of 52,180 mother-child pairs of children within the ages of 6–23 months and mothers aged 15–49 years were included in this study (Table 1)

**No (11):** We have incorporated this comment while preparing the revised manuscript.

**No (12):** Thank you this comment related to science. Generally, one can conduct the outcome with missing data by including in the analysis through **multiple imputations** to handling missing data on the outcome. But in our study prior to statistical analysis, the data were weighted using sampling weight, primary sampling unit, and strata to restore the survey's representativeness and provide accurate statistical estimates.

**Results:**

14. There are several issues in the results section:

- Discrepancies in participant numbers for different countries and missing data (Line 278, Table 2).
- There is discrepancies such as dietary diversity data being available for 746 individuals in Swaziland instead of 763, in Gambia 3527 instead of 1172, and in South Africa 867 in instead of 293. This is just few discrepancies as an example. Thus, the manuscript should clarify how the sample size for each country was determined and address the potential bias introduced by these variations.
- The reference error "Error ! Reference source not found" needs clarification.
- Missing data in baseline variables should be explained, along with the methods used to address the missing data.
- Lines 304 to 308 are better placed in the discussion section.
- Table 4 should include anemia vs. dietary diversity, and a subgroup analysis for different categories of anemia. It is recommended to perform a logistic regression model for anemia vs. MDD, adjusting for various socioeconomic confounders instead of relying on the chi-square results (Table 4).
- The manuscript should clarify the model fit statistics referenced in the text (Lines 313-323). It is not clear why different anemia levels are being compared.

**Response:** Thank you very much for given us several issues under result**.** Simply the data were weighted using sampling weight, primary sampling unit, and strata to restore the survey's representativeness and provide accurate statistical estimates for each county. All issues raised by reviewer were incorporated while preparing a revised manuscript. The odds ratios for the final model appeared to have maintained constant across all cut-off points for the presence of childhood anemia, according to the Brant test of parallel odds assumption (p-value = 0.08 at a 5% level of significance). As a result, the odds ratio results from the models used to compare anemic to non-anemic and severely anemic to mildly/moderately/non-anemic had the same interpretations.

**Discussion:**

15. The discussion should be refocused on the primary study objective, which is the association between anemia levels and MDD. The manuscript appears to emphasize individual significant results of secondary predictors instead.

- While the manuscript mentions the large sample size as a strength indicating there is adequate power to detect the true effect of the independent variable. However, it does not provide a power calculation to support this claim.
- Some limitations are discussed, but the limitation related to the 24-hour recall for minimum dietary diversity is not mentioned and there is potential social desirability bias that could impact on the results. Discuss finding should be interpreted with caution.

**Response:** Thanks for those comments related to reality. So by taking those comments we have incorporates the issue while preparing the revised manuscript as well. Also we have mentioned the limitation as ‘Also, the limitations of our study include the fact that cross-sectional designs cannot completely eliminate recall errors regarding questionnaire information’.

**Recommendation**

This comprehensive report outlines the issues identified throughout the manuscript, with specific line references and requires substantial revisions to address these concerns and enhance the clarity, relevance, and presentation of results. After making the necessary revisions, a reevaluation is recommended for potential publication.
