# Supplementary material for: The effect of dietary diversity on anemia levels among children 6–23 months in sub-Saharan Africa: A multilevel ordinal logistic regression model
Source: PLoS One. 2024 May 21;19(5):e0298647. doi: 10.1371/journal.pone.0298647 (PMC11108208; doi:10.1371/journal.pone.0298647)
Supplement: Attachment — Submitted filename: strobe_plos_one.docx [file pone.0298647.s005.docx]

**Title and Abstract:**

- Address minor grammar issues in lines 28 and 29-32.
- Refine lines 27-32 for clarity.
- Considering that many readers may not go through the entire paper, it is crucial to include odds ratios and 95% CI for lines 48-54, ensuring well-reported results in the abstract.

**Introduction:**

- A reference is needed for lines 99-102.
- Move lines 105-113 to the study strengths in the discussion section.
- Ethical considerations are better suited for inclusion in line 142 and may depend on the specific format requirements of the journal.

**Methods:**

- Clarify the rationale for using a subset of DHS samples (52,180 participants for 32 countries). Consider using all available data (more than 52,180 participants). Provide a clear rationale for excluding some participants, specifying the criteria used for participant selection to address potential bias.
- Explain how missing data were addressed. Clarify that the analysis was based on a complete case and discuss the implications of this in the strength and weakness section of the discussion. Survey weights are not designed to correct for missing data; rather, they account for the sampling distribution during data collection.

**Results:**

- Clarify the error "Error! Reference source not found."
- Report only the model with the best fit and present it in your tables. Remove irrelevant results, such as the null model and model 2, from the tables. Instead, focus on reporting model 3 if that is the model with the best fit. This will enhance clarity and reader comprehension.

Recommendation:

- Overall, once these minor adjustments are made, your paper will be ready for publication. Additionally, note that the paper will require editing, and I expect that PLOS ONE will undertake this process once accepted for publication.
