# Supplementary material for: The effect of dietary diversity on anemia levels among children 6–23 months in sub-Saharan Africa: A multilevel ordinal logistic regression model
Source: PLoS One. 2024 May 21;19(5):e0298647. doi: 10.1371/journal.pone.0298647 (PMC11108208; doi:10.1371/journal.pone.0298647)
Supplement: Attachment — Submitted filename: Response to Reviewers.docx [file pone.0298647.s006.docx]

**Reviewer 3: Comments and response**

We thank you for spending your golden time and providing constructive comments on this manuscript.

**Title and Abstract:**

- Address minor grammar issues in lines 28 and 29-32.

**Response:** Thanks for this helpful comment related to the grammar issue, and then by considering this comment we have incorporated while preparing revised manuscript

- Refine lines 27-32 for clarity.

**Response:** We have incorporated while preparing revised manuscript

- Considering that many readers may not go through the entire paper, it is crucial to include odds ratios and 95% CI for lines 48-54, ensuring well-reported results in the abstract.

**Response:** Great thanks for this helpful comment related to well reported results, and then by considering this comment we have included odds ratios with 95%CI and incorporated while preparing revised manuscript

**Introduction:**

- A reference is needed for lines 99-102.

**Response:** We thank you for your helpful comment related to reference needed, and then by considering this comment we have cited and incorporated while preparing revised manuscript.

- Move lines 105-113 to the study strengths in the discussion section.

**Response:** We thank you for your helpful comment, and then by considering this comment we have incorporated while preparing revised manuscript.

- Ethical considerations are better suited for inclusion in line 142 and may depend on the specific format requirements of the journal.

**Response:** We thank you for your comment, based on the specific format requirements of the journal we included at the end of the method section (lines 250-254) and then by considering this comment we have incorporated while preparing revised manuscript

**Methods:**

- Clarify the rationale for using a subset of DHS samples (52,180 participants for 32 countries). Consider using all available data (more than 52,180 participants). Provide a clear rationale for excluding some participants, specifying the criteria used for participant selection to address potential bias.

**Response:** We thank you for your useful comment related to rationality of selecting a sample, and then by considering this comment we have incorporated while preparing revised manuscript (lines 133-139).

- Explain how missing data were addressed. Clarify that the analysis was based on a complete case and discuss the implications of this in the strength and weakness section of the discussion. Survey weights are not designed to correct for missing data; rather, they account for the sampling distribution during data collection.

**Response:** We thank you for your useful comment related to explain how the missing data was addressed, and then by considering this comment we have incorporated while preparing revised manuscript (lines 511-528).

**Results:**

- Clarify the error "Error! Reference source not found."

**Response:** We thank you for your comment. But dear reviewer, we are not seeing any “Error! Reference source not found” under results and if you see any “Error” please let us know where it is specifically and we will fix it. If it is related to figures, we have entered the figures separately as journal guideline. We put only the figures tittle in the document. And again, let us know the (Lines__) that says “Error! Reference source not found”.

- Report only the model with the best fit and present it in your tables. Remove irrelevant results, such as the null model and model 2, from the tables. Instead, focus on reporting model 3 if that is the model with the best fit. This will enhance clarity and reader comprehension.

**Response:** We thank you for your suggestions and comments. Having this, from our point of view, we have the idea that all models (Null model, Model I, Model II, and Model III) should be present so that we can clearly understand the results of null model (result of rando effect), Model I (result of individual level), Model II (result of community level) and Model III (result of individual and community level jointly). But dear reviewer, if you don’t believe it and you need to change it, we are willing to change it again.

**Recommendation:**

- Overall, once these minor adjustments are made, your paper will be ready for publication. Additionally, note that the paper will require editing, and I expect that PLOS ONE will undertake this process once accepted for publication.
